# Adaptive immune responses and cytokine immune profiles in humans following prime and boost vaccination with the SARS-CoV-2 CoronaVac vaccine

**DOI:** 10.1186/s12985-022-01957-1

**Published:** 2022-12-22

**Authors:** Chan Wang, Songhao Yang, Liangwei Duan, Xiancai Du, Jia Tao, Yana Wang, Jihui Yang, Yongxue Lv, Junliang Li, Cuiying Zhang, Jia Wen, Yazhou Zhu, Liangliang Chang, Hui Wang, Qi Wang, Wei Zhao

**Affiliations:** 1 School of Basic Medicine, Ningxia Medical University, Yinchuan, 750004 Ningxia Hui Autonomous Region People’s Republic of China; 2 Key Laboratory of Hydatid Disease of Ningxia Medical University, Yinchuan, 750004 Ningxia Hui Autonomous Region People’s Republic of China; 3 Center of Scientific Technology of Ningxia Medical University, Yinchuan, 750004 Ningxia Hui Autonomous Region People’s Republic of China; 4 Henan Key Laboratory of Immunology and Targeted Drugs, School of Laboratory Medicine, Xinxiang Medical University, Xinxiang, China

**Keywords:** SARS-CoV-2, Inactivated vaccine, CD4^+^T cells, CD8^+^T cells, Memory B cells

## Abstract

**Background:**

Adaptive immune response has been thought to play a key role in SARS-CoV-2 infection. The role of B cells, CD4^+^T, and CD8^+^T cells are different in vaccine-induced immune response, thus it is imperative to explore the functions and kinetics of adaptive immune response. We collected blood samples from unvaccinated and vaccinated individuals. To assess the mechanisms contributing to protective immunity of CoronaVac vaccines, we mapped the kinetics and durability of humoral and cellular immune responses after primary and boost vaccination with CoronaVac vaccine in different timepoints.

**Materials and methods:**

We separate PBMC and plasma from blood samples. The differentiation and function of RBD-spcific CD4^+^T and CD8^+^T cells were analyzed by flow cytometry and ELISA. Antibodies response was analyzed by ELISA. ELISPOT analysis was perfomed to detected the RBD-spcific memory B cells. CBA analysis was performed to detected the cytokine immune profiles. Graphpad prism 8 and Origin 2021 were used for statistical analysis.

**Results:**

Vaccine-induced CD4^+^T cell responses to RBD were more prominent than CD8^+^T cell responses, and characterized by a predominant Th1 and weak Th17 helper response. CoronaVac vaccine triggered predominant IgG1 antibody response and effectively recalled specific antibodies to RBD protein after booster vaccination. Robust antigen-specific memory B cells were detected (*p* < 0.0001) following booster vaccination and maintained at 6 months (*p* < 0.0001) following primary vaccination. Vaccine-induced CD4^+^T cells correlated with CD8^+^T cells (r = 0.7147, 0.3258, *p* < 0.0001, *p* = 0.04), memory B cell responses (r = 0.7083, *p* < 0.0001), and IgG and IgA (r = 0.6168, 0.5519, *p* = 0.0006, 0.003) after vaccination. In addition, vaccine induced a broader and complex cytokine pattern in plasma at early stage.

**Conclusion:**

Taken together, these results highlight the potential role of B cell and T cell responses in vaccine-induced long-term immunity.

## Introduction

An unprecedented worldwide pandemic caused by SARS-CoV-2 has killed more than 6 million people worldwide [1]. Vaccination as the most effective prophylaxis measure is imperative [2–4]. Many countries have approved the use of inactivated vaccines [5, 6]. Inactivated vaccines have been widely used since ancient times [7]. CoronaVac that was a β-propyl lactone-inactivated SARS-CoV-2 vaccine developed by Sinovac Life Sciences in China and generated protective efficacy up to 79.34% [8–10]. Most previously published reports on CoronaVac vaccine have focused on vaccine-induced neutralizing antibidies [11–13]. However, the adaptive immune system is important for control of most viral infections. So far, studies associated with vaccine-induced immune protection for SARS-CoV-2 remain varied, with data from mRNA and adenovirus vaccines indicating the involvement of both cellular and humoral mechanisms [14, 15].

Growing evidence suggests that T cell response, particularly CD4^+^T cells play key role in defending against viral infections and may induce long-term immune responses [16–18]. Data from cilinical studies showed that the loss of CD4^+^T cells responses were significantly related to disease severity in COVID-19 patients [19]. In addition, antigen-specific T cells were detected in SARS-CoV patients who had been infected for several years, suggesting an important role for antigen-specific T cells in generating lasting immunity against viruses [20]. A balanced humoral and Th1-type cellular immune response may be important for the prevention of COVID-19 and the development of effective vaccine-induced immunity [21, 22]. Data demonstrated that a predominant Th1-type response was detected in mRNA and adenovirus-vectored individuals [20, 23]. Little is known about the kinetics of priming for vaccine-induced CD4^+^T and CD8^+^T cells in the context of CoronaVac vaccination.

Clinical data showed that vaccine-induced antibody levels gradually decreased over time [24], and the durable protective effect induced by vaccines still needs long-term attention. Memory B cells are important components of long-lasting humoral immune memory [25]. The effective induction of longevous memory B cells is critical to preventing virus infection and protecting against re-exposure [26–28]. Studies of COVID-19 patients have suggested memory B cells were durable for over eight months post-infection [28]. In the context of mRNA vaccination, remarkable B cell activation and proliferation was detected [26, 29]. Therefore, prediction of vaccine efficacy should not solely rely on NAbs titers. Rather, memory B cells should be taken into account.

Virus infections induce a proinflammatory response including expression of cytokines [30]. Cytokines play central roles in the host response to viral infections as well as in the immunopathology associated with many viral diseases [31]. Therefore, assessing cytokine immune profiles induced by vaccine may be better characterize the immune response induced by the vaccine.

In this study, we collected blood samples from CoronaVac vaccination individuals in Ningxia on day 40, 180 after two dose vaccination, and on day 60 after booster vaccination. To assess the mechanisms contributing to protective immunity of CoronaVac vaccines, we mapped the kinetics of vaccine-induced antibodies, memory B cells, the differentiation and function of RBD-spcific CD4^+^T and CD8^+^T cells, and cytokine profile in plasma. Finally, we assessed the relation between cellular response and humoral response. This study will provide reference data for further research on inactivated vaccine based vaccination protocols to produce higher immune efficacy.

## Materials and methods

### Human subjects

Sixty-four individuals (35 unvaccinated donors, 29 vaccinated donors) agreed enrolled in the study and were approved by the Institutional Review Committee of Ningxia Medical University. They were negative for specific antibodies to SARS-CoV-2 RBD protein and reported no prior history of COVID-19 or being positive for SARS-CoV-2 infection. These participants had no history of major systemic diseases such as autoimmune diseases, congestive heart failure, hepatitis B or C or HIV and were considerd healthy. Written informed consent was obtained from all participants. All donor samples were collected between 2021 and early 2022. Blood samples were collected at four different time points: before vaccination (unvaccinated, n = 35), day 40 post the 2nd dose (40d dose2, n = 29), six months post the 2nd dose (180d dose2, n = 29), and day 60 post the 3rd dose (60d dose3, n = 29). Vaccinated donors received a dosage of 600 SU/0.5 mL CoronaVac vaccines on days 0 and 38 and received the 3rd dose vaccine 6 months after the second vaccination. The characteristics of these participants were presented in Table 1.

**Table 1:** Participant characteristics

Age (years)	Unvaccinated (n = 35) 19–77 (median = 30, IQR = 17.5)	Vaccination (n = 29) 19–62 (median = 27, IQR = 13.5)
*Gender*		
Male (%)	49% (17/35)	48% (14/29)
Female (%)	51% (18/35)	52% (15/29)
*Past medical history*		
No known	N/A	N/A
Hyperlipidemia	9% (3/35)	7% (2/29)
Hypertension	N/A	7% (2/29)
Asthma	N/A	N/A
Known or suspected sick contact/exposure	N/A	N/A
*Residency*		
Ningxia (%)	100% (35/35)	100% (29/29)
Antibody test positivity	N/A	N/A

### Preparation of PBMCs and plasma

Whole blood obtained from heparinised venous blood was left undisturbed at 23 °C for 30 min. Blood samples were centrifuged at 450 g for 5 min to separate PBMC and plasma. Plasma was subpacked and stored at – 80 °C. PBMCs were obtained by Ficoll (TBD, LTS1077) after 1:1 dilution in Hank’s. Red blood cells in PBMC were removed by red blood cell lysis buffer (Solarbio, China). PBMCs were stored in serum-free cell freezing medium (NCM, C40050) in liquid nitrogen if not immediately used for the downstream process.

### ELISA for estimating RBD protein-specific antibodies

ELISA was conducted to determine the antibodies and titres of serum binding antibodies to SARSCoV-2 RBD. Corning 96-well Stripwell Flat Bottom Microplates (Corning® 9102) were coated with 2.5 μg/mL SARS-CoV-2 RBD protein overnight at 4 °C. Plates were washed 5 times the next day with PBST (PBS containing 0.05% Tween-20) to remove unbound RBD protein and then blocked with 5% skim milk (Biotopped, D6340) in PBST for 2 h at 37 °C. For tilter, twofold serially diluted plasma were added to the wells and incubated for 1 h at 37 °C. For RBD specific antibodies, plasma was added to the wells after 1:500 dilution in 5% milk and incubated for 1 h at 37 °C. For IgG, Rb pAb to Hu IgG (HRP) antibody (abcam, ab6759) was used at a 1:10,000 dilution. For IgM, Rb pAb to Hu IgM (HRP) antibody (abcam, ab97210) was used at a 1:8000 dilution. For IgA, Rb pAb to Hu IgA (HRP) antibody (abcam, ab73901) was used at a 1:2000 dilution. For IgG subsets, Mouse anti-human IgG1-4 Fc secondary antibody (nitrogen, MH1715, MH1722, MH1732, MH1742) was used at a 1:200 dilution. Plates were washed 5 times with PBST. Plates were developed with TMB Two-component Substrate solution (solarbio, PR1210) for 5–30 min at room temperature. The reaction was stopped with ELISA stop solution. Plates were read on a Spectramax Plate Reader at 450 nm using Thermo Scientific Multiskan SkyHigh.

### Flow cytometry

PBMCs (5 × 10^5^) were resuspended in 100 μL of Buffer2 (as prevous reported [32]) for the surface stain. Then, the surface markers were added to stain the cells for half-hour at 4 °C, protected from light. For intracellular cytokine staining, samples were then fixed for 8 min protecting from light using 4% paraformaldehyde and permeabilised for 2 h in the dark using Buffer2 (as prevous reported [32]). After washing, the cells were stained using intranuclear antibodies in the dark for half-hour at 4 °C. 300 μL of Buffer2 was added to the cells. The data was analysed using the program FlowJo version 10.0. All antibodies are shown in Table 2.

**Table 2:** Antibodies

Antibody	Conjugation	Clone	Source	Catalog number
CD3	AF700	SK7	Biolegend	344,822
CD4	BV605	OKT4	Biolegend	317,438
CD8	Percp5.5	SK1	Biolegend	344,710
OX40	BV510	Ber-AC735	Biolegend	350,026
4-1BB	BV421	4B4-1	Biolegend	309,820
CD69	PE	FN50	Biolegend	310,906
TNF	APC	Mab11	Biolegend	502,912
IFN	BV510	45B3	Biolegend	502,912
IL-2	BV421	MQ1-17H12	Biolegend	500,307
IL17A	FITC	BL168	Biolegend	512,330
IL-4	PE	MP4-25D2	Biolegend	500,826

### Cell stimulation

For intracellular cytokines, cells were stimulated by 10 μg/mL SARS-CoV-2 specific RBD protein for 24 h in 48-well plates. Cells were diluted into 1 × 10^6^ PBMC per well. BFA (Solarbio) was added into stimulated cells in the last 6 h of incubation. Following a twenty-four hours stimulation, the cells were collected and used for intracellular cytokine staining.

For the AIM assay, cells were co-cultured with 10 μg/mL of SARS-CoV-2 RBD protein for six hours in 1 μg/mL of purified NA/LE Mouse anti-human CD28 antibody (BD Biosciences, 555,725). Positive controls were performed with 1 μg/mL of PHA (Thermo Fisher Scientific, 10,576,015). Anti-CD4, anti-CD8, anti-4-1BB, anti-OX40, and anti-CD69 antibodise were added to the cells suspension. Cells were washed in Buffer2 and added 300 μL of Buffer2 into cells for flow cytometry.

### ELISA for detecting cytokines

Cells were co-cultured with 10 μg/mL of SARS-CoV-2 RBD protein for six hours in the presence of 1 μg/mL of purified NA/LE Mouse anti-human CD28 antibody. After incubation for 6 h, ELISA was used to detected the supernatants cytokines including IFN-γ, TNF-α, IL-2, IL-17A, and IL-4 according to the manufacturer's protocol (BD Biosciences).

### SARS-CoV-2 specific memory B cell ELISPOT assay

PBMCs were cultured at 1.5 × 10^6^ cells/well in RPMI 1640 medium (GIBCO) supplemented with a cultural medium in 48-well plates alone or with Human Memory B-cell Stimpack (Mabtech, USA), including the TLR7/8 agonist R848 (1 μg/mL) and recombinant human IL-2 (10 ng/mL).

After incubation for 5 days, cells were harvested, washed with Hank’s, diluted into 5 × 10^5^/well in completed medium, and finally plated on prepared ELISPOT plates. 10 μg/mL SARS-CoV-2 RBD antigen was coated on 96-well filtration plate (Mabtech, USA) overnight at 4 °C and washed thrice with a 10% FBS RPMI medium. Then plates were blocked by 10% FBS RPMI medium for two hours at 23 °C. Cells from 5-day cultures were plated in ELISPOT plates in the culture media described above at concentrations of 5 × 10^5^ cells/well to detect SARS-CoV-2 RBD specific IgG^+^ ASCs. After a 24 h incubation in a 5% CO_2_ incubator, firstly, the plate was washed twice with ddH_2_O and then washed thrice with PBST. Monoclonal antibody to human IgG (Mabtech, USA) diluted 1:200 using PBS with 10% FBS was added to wells and incubated for two hours in 37 °C incubator box. Plates were washed thrice with PBST. Next, streptavidin-HRP diluted to 1:1000 in PBS with 10% FBS was added to wells and incubated for 1 h at 37 °C. Plates were first washed four times using PBST and then washed twice using PBS. Color developed was used with AEC Substrate Set (BD) for 5–30 min at 23 °C. ddH_2_O was used to terminate the reaction. Results were analysed using AID ELISpot Reader Classic.

### Cytometric bead array for estimating cytokine immune profiles

The Cytometric Bead Array Human Th1/Th2/Th17 Cytokine Kit and the Inflammatory Cytokines Kit (BD) were used to detecte the cytokines in plasma according to the manufacturer’s instruction. In simple terms, beads coated with capture antibodies response to IL-17A, IFN-γ, TNF-α, IL-10, IL-6, IL-4, IL-2, IL-12p70, IL-1β, and IL-8 were added to 50 µL plasmas and incubated in a 12 × 75-mm tube in the dark for 1.5 h at 23 °C. Added 1 ml of wash buffer into each test tube and centrifuged at 200 g for 5 min. 50 µL of cytokine PE Detection Reagent was added to the mixture and incubated for 1.5 h at 23 °C. Finally, the sample was washed and analyzed on the flow cytometer. The samples were analysed using FACS Array software.

### Statistical analysis

Graphpad prism 8 and Origin 2021 were used for statistical analysis. Data were presented as means ± standard deviations. Comparing ratio differences between two groups used Wilcoxon Tests. Multiple comparisons used Kruskal–Wallis and Dunn’s post-test. Spearman’s rank correlation was used to analyse correlation. Statistical significance was considered *p* ≤ 0.05.

## Results

### ***Prime-boost vaccination elicited RBD-specific CD4***^+^***T and CD8***^+^***T cells***

**Fig. 1:**
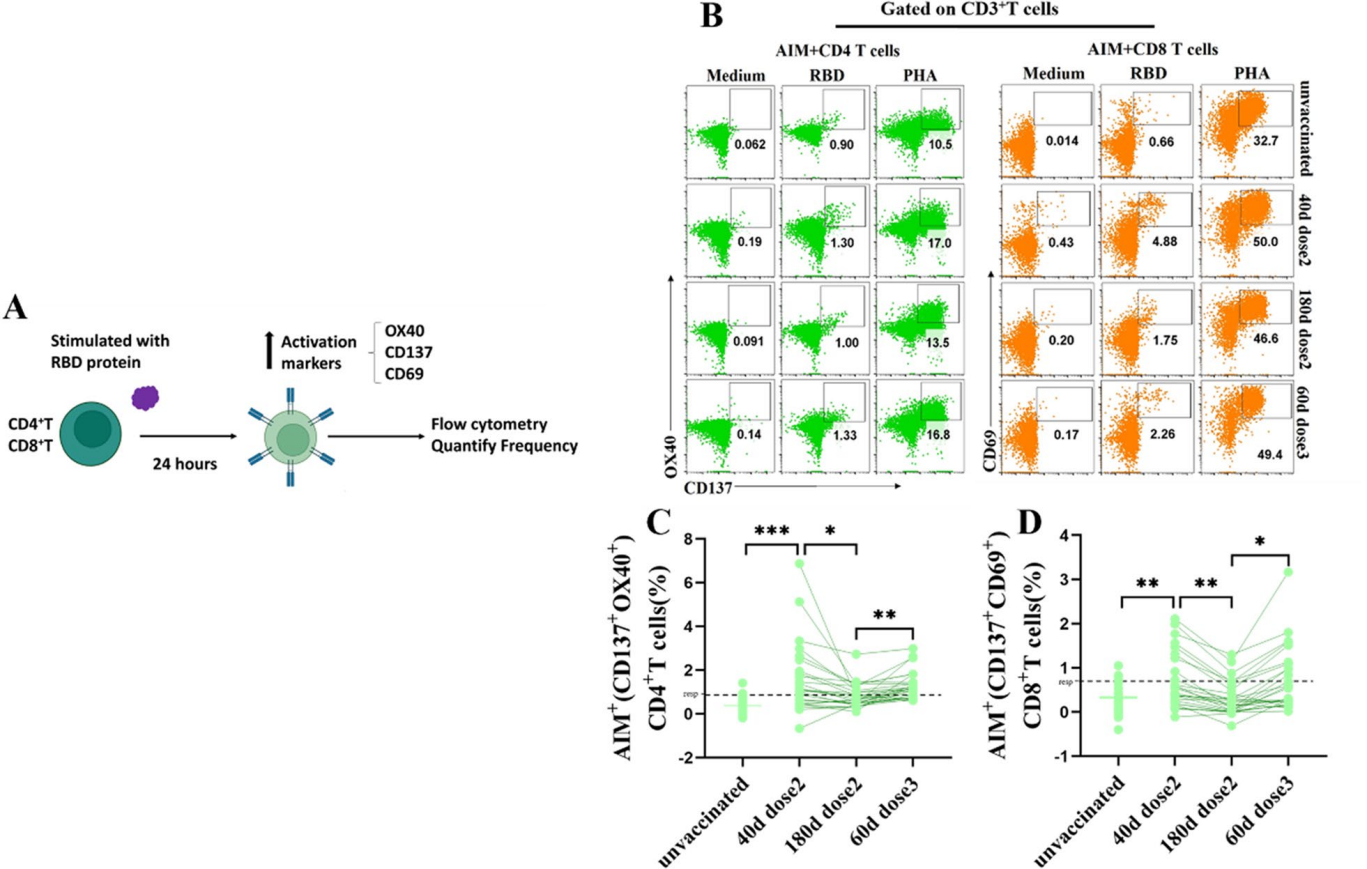
Prime-boost vaccination elicited RBD-specific CD4^+^T and CD8^+^T cells. **A** Experimental design for AIM. **B** Representative flow cytometry plots for RBD-specific CD4 (left) and CD8^+^T cell (right) after stimulation of PBMCs with RBD protein, gated on CD3^+^CD4^+^T cells. The scatter in the frame represented the frequency of AIM^+^CD4 and CD8^+^T. **C** Proportions of AIM^+^CD4 (left) and CD8^+^T (right). responder (resp) cut-off is 0.84 (AIM^+^CD4) and 0.80 (AIM^+^CD8) (dotted line). *: *P* < 0.05, **: *P* < 0.01, ***: *P* < 0.001, ns: no significance. Significant difference was calculated by Kruskal–Wallis’s test

The RBD-specific CD4^+^T and CD8^+^T cells responses were measured with a flow cytometry T cell receptor (TCR) dependent activation-induced marker (AIM) assay using SARS-CoV-2 RBD protein. We found that a prominent increase in AIM^+^(CD137^+^OX40^+^) CD4^+^T cells on day 40 following prime vaccination and stronger on day 60 after booster vaccination (*p* = 0.0001, *p* < 0.0001, Fig. 1B, C). The frequency of AIM^+^CD4^+^T cells on day 180 (six months) after prime vaccination was significantly decreased (*p* = 0.02). We found a similar pattern with AIM^+^(CD69^+^CD137^+^) CD8^+^T cells after vaccination (*p* = 0.01, *p* = 0.03, Fig.1B, D). However, the frequency of the AIM^+^CD4^+^T cells at each study timepoint remained significantly higher than that of AIM^+^CD8^+^T cells. In agreement with previous studies [27, 28], we detected AIM^+^CD4^+^T and CD8^+^T cells in 6% and 10% of unvaccinated individuals, respectively, which may be attributed to cross-reactive T cells that were probably generated during previous encounters with seasonal coronaviruses. In general, CoronaVac vaccine induced the activities of RBD-specific CD4 and CD8^+^T cells following prime and boost vaccination.

### ***CoronaVac vaccine triggered polyfunctional CD4***^+^***T cells response with a predominant Th1 and weak Th17 helper response against RBD protein***

**Fig. 2:**
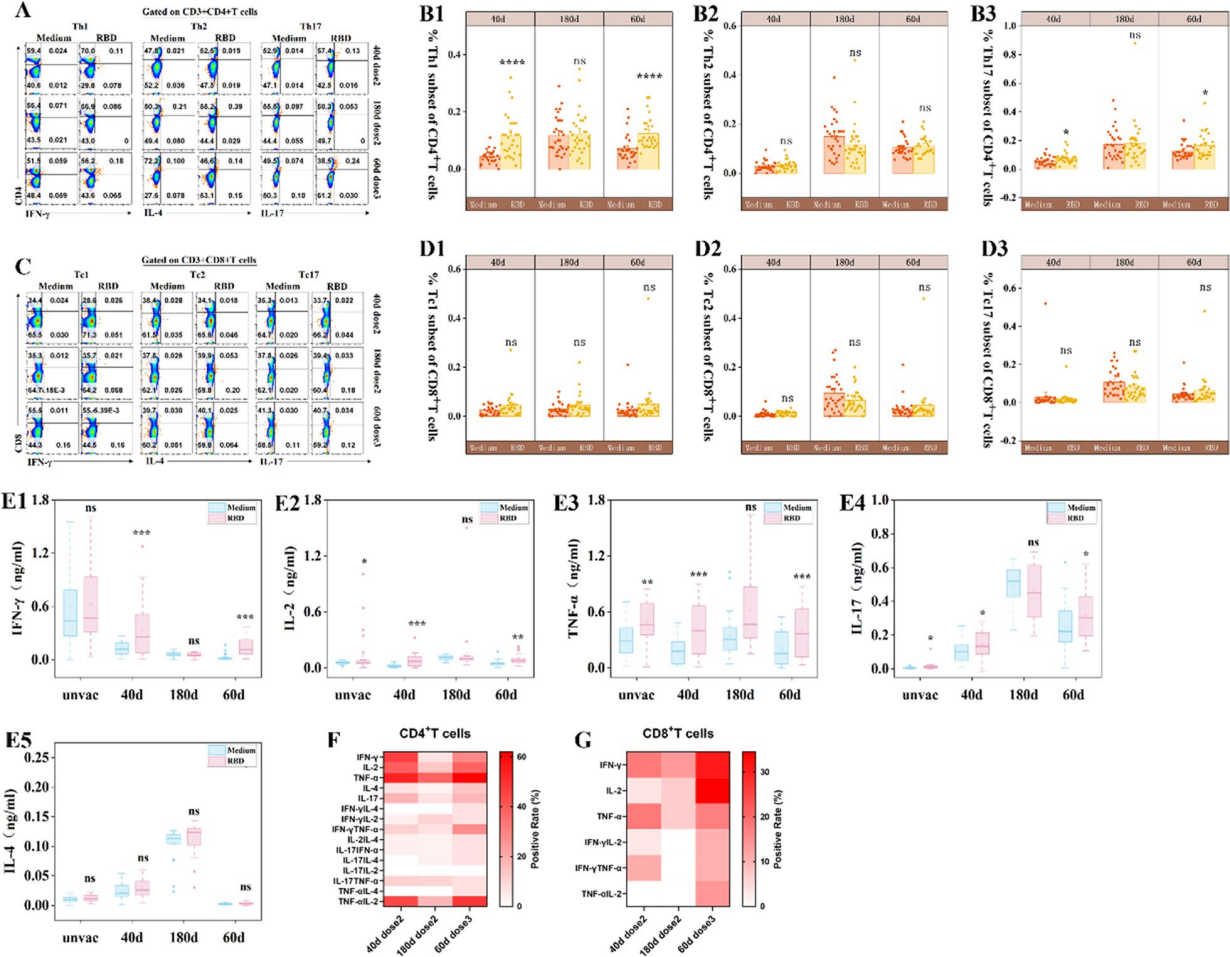
CoronaVac vaccine triggered polyfunctional CD4^+^T cells response with a predominant Th1 and weak Th17 helper response against RBD protein. **A, C** Representative flow cytometric plots depicting the gating of CD4 and CD8^+^T cells subset. **B, D** Frequency of subsets in CD4 and CD8^+^T cells. **E** ELISA detected the cytokines from cultural supernatant. **F, G** Heat map of multifunctional activity profiles of the RBD-specific CD4 and CD8^+^T cells evaluated on days 40, 180 (two doses) and 60 (three doses). *: *P* < 0.05, **: *P* < 0.01, ***: *P* < 0.001, ns: no significance. Significant difference was calculated by Wilcoxon tests [(B), (D), (E)], and Kruskal–Wallis’s test [(F)]

To assess functionality and polarization of the RBD-specific CD4 and CD8^+^T cells responses after prime and boost vaccination, we measured by intracellular cytokine staining (ICS) analysis in response to a 24 h stimulation of PBMC with RBD protein. For CD4^+^T cell polarization we observed dominant Th1 cells and modestly weaker induction of Th17 cells on day 40 after the prime two dose of CoronaVac vaccine (*p* < 0.000, *p* = 0.04, Fig. 2B1, B3). The same phenomenon was observed following booster vaccination on day 60 (*p* < 0.000, *p* = 0.04). However, we did not observed a polarization of CD8^+^T cells (*p* > 0.05, Fig. 2C, D). We measured the cytokines secreted into the supernatant by ELISA. The result showed that vaccine led to a robust increase in IFN-γ, IL-2 and TNF-α levels (*p* = 0.003, *p* < 0.0001, *p* = 0.0003, Fig. 2E1–E3) and modestly increased the level of IL-17A (*p* = 0.03, Fig. 2E4) on day 40 and 60 compared to controls. Cytokine IL-4 did not generated remarkable difference after vaccination (*p* > 0.05, Fig. 2E5). Additionally, we detected a few cells expressed TNF-α, IL-2, and IL-17A (*p* = 0.004, *p* = 0.03, Fig. 2E2–E4) in unvaccinated individuas, indicating may be exist the preexisting cross-reactive memory to SARS-CoV-2.

To qualitatively assess RBD-specific CD4 and CD8^+^T cells for polyfunctional responses after prime and boost vaccination, we performed coexpression analysis using Boolean gating. Dominant RBD-specific CD4^+^T and CD8^+^T cells on day 40 and 60 expressed IFN-γ, IL-2, or TNF-α alone or combination with each other (Fig. 2F, G). A small CD4^+^T cell groups expressed IL-17, IL-4, or both. On day 180, the proportion of CD4^+^T cells expressing one and two cytokines remarkable decreased. However, response on day 180 dominated by CD8^+^T cells expressing single cytokines. The function of coexpressing two cytokines was more stronger in the CD4^+^T cells than the CD8^+^T cells. In summary, these results demonstrated that CoronaVac vaccine mainly induced functional CD4^+^T cell responses in most vaccination individuals after prime and boost vaccination, with a predominant Th1 and a weak Th17 polarization of the helper response.

### CoronaVac vaccine triggered predominant IgG1 antibody response and effectively recalled specific antibodies to RBD protein after booster vaccination

**Fig. 3:**
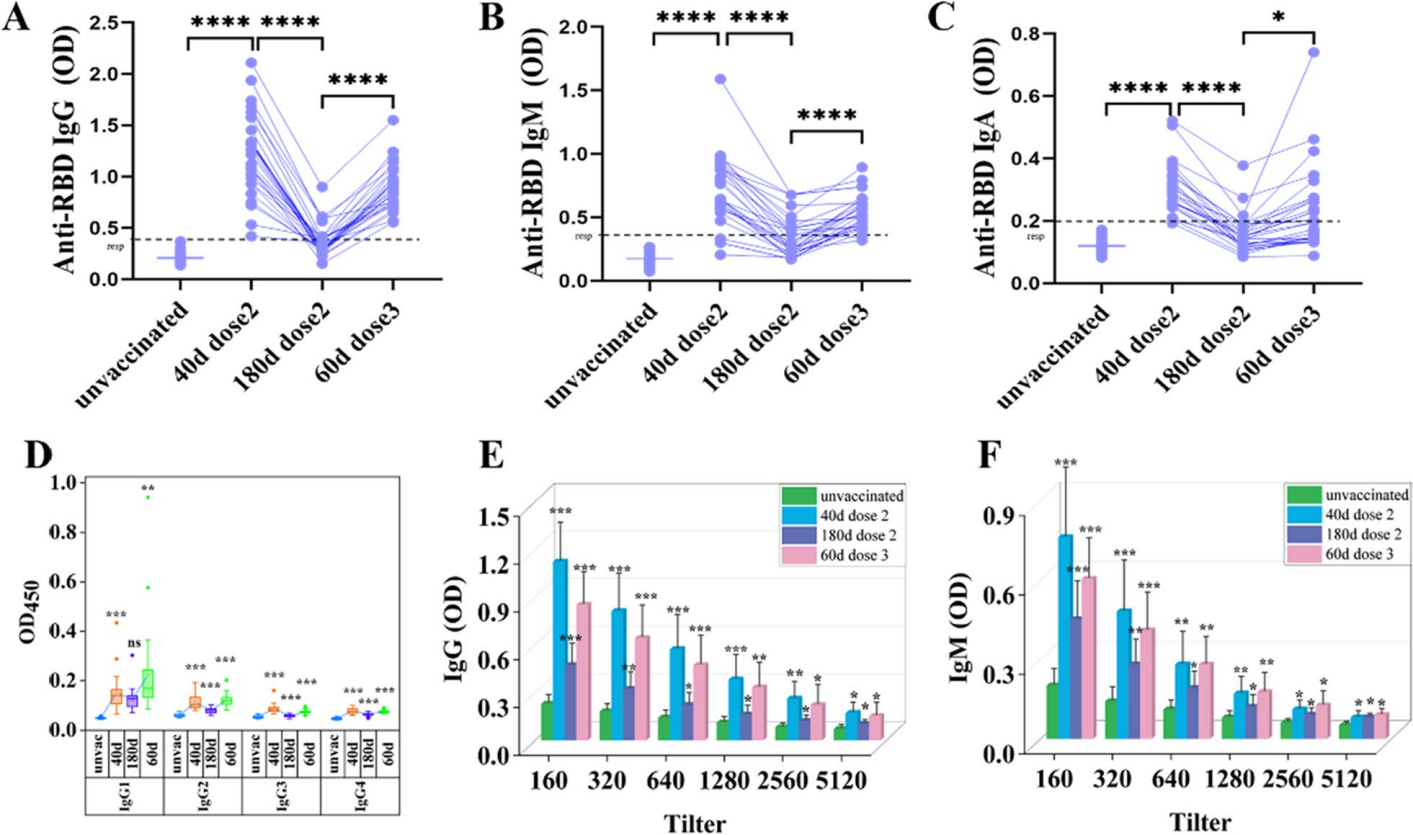
CoronaVac vaccine triggered predominant IgG1 antibody response and effectively recalled specific antibodies to RBD protein after booster vaccination. **A–D** ELISA for **A** IgG, **B** IgM, **C** IgA, and **D** IgG subclass from 35 unvaccinated donors and 29 vaccinated donors. responder (resp) cut-off is 0.39 (IgG), 0.34 (IgM), and 0.2 (IgA) (dotted line). **E, F** Plasma IgG and IgM ELISA titers to SARS-CoV-2 RBD protein. *: *P* < 0.05, **: *P* < 0.01, ***: *P* < 0.001, ns: no significance. Significant difference was calculated by Wilcoxon tests

In order to study the SARS-CoV-2 RBD-specific antibodies responses following prime-boost CoronaVac vaccine. We detected RBD-specific IgG, IgM, IgA, and IgG subsets antibodies responses. Anti-RBD IgG and IgM antibodies were detected in 100% (29/29) (Fig. 3A) and 83% (24/29) (Fig. 3B) of subjects on day 40 and the levels were remarkable reduction up to 6 months after the 2nd dose vaccination, for 28% (8/29) and 38% (11/29) of subjects. These responses rates increased to 100% (29/29) and 93% (27/29) after booster vaccination. IgA antibody were detected in 83% (24/29) of subjects on day 40 after the 2nd dose vaccination (Fig. 3C). However, it's almost undetectable up to 6 months after 2nd dose vaccination. This response rate increased to 48% (14/29) after booster vaccination. In addition, we analyzed the IgG subclass against SARS-CoV-2 RBD protein (Fig. 3D). The IgG1 subclass was detected as the major antibody subclass after vaccination. The titers of RBD-IgG and IgM were categorized as 1:80, 1:160, 1:320, 1:640, 1:1280, 1:2560, and 1:5120. Titers less than 1:80 are considered as negative, 1:80–1:160 as low titers, 1:320–1:640 as moderate titers, and 1:1280 and ≥ 1:2560 as high titers [33]. As shown in Fig. 3E, F, the titers of RBD-IgG and IgM on day 40 after the 2nd dose vaccination displayed high titers (*p* < 0.0001). However, there was a remarkable reduction up to 6 months after the 2nd dose vaccination and the titers still presented high titers (*p* < 0.0001, *p* = 0.001). Booster vaccination obviously increased the IgG and IgM tilters (*p* < 0.0001, *p* = 0.001). These results demonstrated CoronaVac vaccine triggered predominant IgG1 antibody response and effectively recalled specific antibodies to RBD protein after booster vaccination.

### Elicitation of robust memory B cell responses to SARS-CoV-2 RBD protein following booster vaccination

**Fig. 4:**
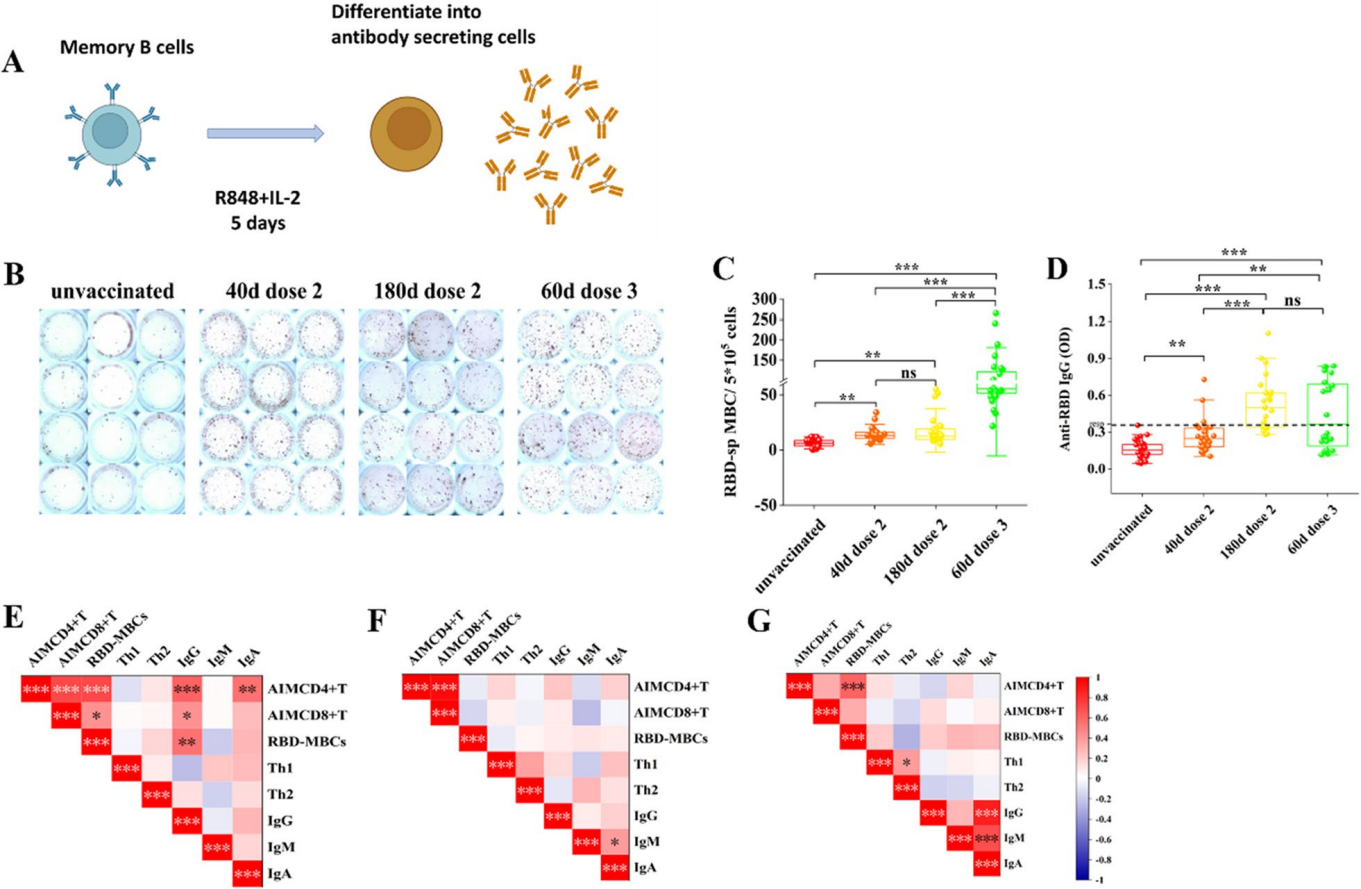
The kinetic of memory B cell responses to SARS-CoV-2 RBD protein and correlation with RBD-specific T cell responses after vaccination. **A** Experimental design for differentiation of memory B cells into antibody secreting cells. **B, C** Comparative memory B cells ELISpot spot forming units (SFUs) per 5 × 10^5^ PBMC in individuals with unvaccination, and vaccination with SARS-CoV-2 RBD. **D** ELISA detected anti-RBD IgG secreted by RBD-specific memory B cells from cultural supernatant. responder (resp) cut-off is 0.36 (dotted line). **E–G** Heatmap showing associations among AIM^+^CD4, AIM^+^CD8, Th1, Th2, memory B cells, IgG, IgM, and IgA on day 40 **(E)**, 180 **(F)**, and 60 **(G)** after vaccination. Colours indicated the level of correlation (red– positive, white—no correlation, blue—negative). *: *P* < 0.05, **: *P* < 0.01, ***: *P* < 0.001, ns: no significance. Significant difference was calculated by Kruskal–Wallis’s test

Since maintaining extensive protective antibodies and RBD-specific memory B cells are key features of long-term protective immunity, we evaluated whether vaccine-induced memory B cells can produce effective antibodies after activation. Peripheral blood mononuclear cells (PBMCs) were stimulated with human memory B-cell stimpack to differentiate memory B cells into antibody-secreting cells (ASCs).As the ELISPOT result, the frequency of RBD-specific memory B cells on day 40 following prime vaccination (*p* < 0.0001, Fig. 4B, C) presented a relative increase compared with the unvaccinated cohort and was still detected up to 6 months post-vaccination (*p* < 0.0001). The booster vaccination caused stranger memory B cells response than prime (*p* < 0.0001). We detected the antibody IgG secreted by RBD-specific memory B cells in the cultural supernatant (Fig. 4D). Anti-RBD IgG antibodies were detected in the cultural supernatant from prime (*p* = 0.001, *p* < 0.0001) and booster vaccination cohorts (*p* < 0.0001). This indicated that vaccine-induced memory B cells sustained at least six months after prime vaccination, and booster vaccination was imperative for long-term humoral immune memory to agaist SARS-CoV-2.

### ***Vaccine-induced CD4***^+^***T cell responses correlated with CD8***^+^***T cell and humoral responses***

CD4^+^T cells play an importantly auxiliary role in CD8^+^T and humoral responses. Th1 cells primarily promote CD8^+^T cell responses, whereas Th2 cells help foster humoral immune response. Therefore, we assessed the relationship among AIM^+^CD4, AIM^+^CD8, Th1, Th2, RBD-specific memory B cells, IgG, IgM, and IgA antibosies. We observed a strong correlation between total AIM^+^CD4 and AIM^+^CD8 cells after vaccination (r = 0.7147, 0.3258, *p* < 0.0001, *p* = 0.04, Fig. 4E–G). In addition, the frequency of RBD-specific memory B cells correlated with AIM^+^CD4 cells (r = 0.7083, *p* < 0.0001), however, were less well correlated with AIM^+^CD8 cells on day 40 after prime vaccination (r = 0.4345, *p* = 0.02, Fig. 4E). Notably, we observed that AIM^+^CD4 cells correlated with IgG and IgA (r = 0.6168, 0.5519, *p* = 0.0006, 0.003, Fig. 4E) and RBD-specific memory B cells correlated with IgG (r = 0.2775, *p* = 0.003, Fig. 4E). We also observed strong correlation among IgA, IgG, and IgM after booster vaccination (r = 0.4987, 0.6935, *p* = 0.007, *p* < 0.0001, Fig. 4G). These results indicated that vaccine-induced CD4^+^T cell responses may augment and coordinate the CD8^+^T and humoral responses.

### Elicitation of broad and complicated cytokine immune profiles in plasma at early stage following prime and boost vaccination

**Fig. 5:**
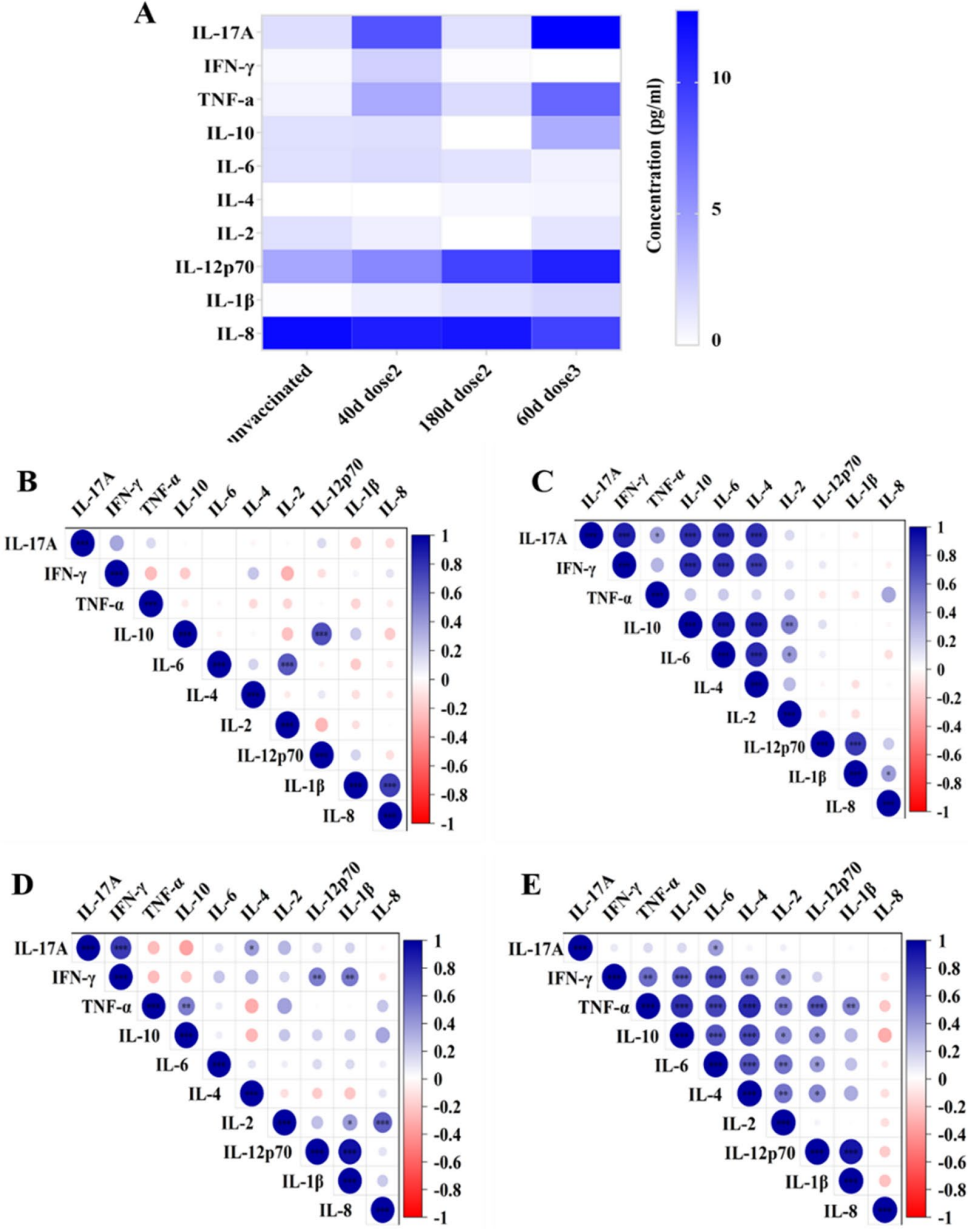
Clustering of net cytokine signatures in response to SARC-CoV-2 inactived vaccination. **A** Th1/Th2/Th17 cytokines and inflammatory levels were measured using the human Cytometric Bead Array in plasma samples. Concentration was presented pg/ml. Cytokine matrix analysis response to the SARS-CoV-2 inactivated vaccine. Correlogram depicted the relationships between cytokines in plasma from the **B** unvaccinated cohort, **C** 40d dose2 cohort, **D** 180d dose2 cohort, and **E** 60d dose3 cohort. Colours indicated the level of correlation (blue—positive, white—no correlation, red—negative)

Cytokine profiles in plasma from subjects after prime and booster vaccination were analysed using the Cytometric Bead Array (CBA). Th1 type cytokines IFN-γ, TNF-α, IL-12p70, IL-2, Th2 type cytokines IL-4, Th17 type cytokines IL-17A, immunoregulation cytokine IL-10, and proinflammatory cytokine IL-6, IL-8 and IL-1β were among the cytokines analysed. IL-8 and IL-12p70 were the most abundantly secreted cytokines from all individuals (Fig. 5A). TNF-α and IL-17A were abundantly secreted cytokines in the 40d dose2 cohort (*p* < 0.0001) and the 60d dose3 cohort (*p* = 0.02, *p* = 0.001), which was consistant with ICS result. IL-12p70, a key cytokine that initiated the Th1 response, was remarkable increased (*p* < 0.0001) following booster vaccination.

The cytokine profile correlation matrix of each group was established to obtain the following results: (1) In the unvaccinated cohort, we did not found correlations among these cytokines (Fig. 5B). (2) The cluster in the 40d dose2 cohort: cluster one consisted of IL-10, IL-6, IL-4, IL-17A, IFN-γ, and TNF-α, while cluster two consisted of IL-10, IL-6, IL-4, and IL-2 (Fig. 5C). (3) In the 180d dose2 cohort, we did not found correlations among these cytokines (Fig. 5D). (4) In the 60d dose3 cohort, a big cluster of cytokines showed correlations. A cluster consisting of IFN-γ, IL-10, IL-6, IL-4, IL-12p70, TNF-α, and IL-2 presented strong correlations (Fig. 5E). These findings indicated that CoronaVac vaccine induced a strong correlation among cytokines at the early stage after prime and booster vaccination, but the correlation and complexity among cytokines gradually decreases over time.

## Discussion

Adaptive immune response has been thought to play a key role in SARS-CoV-2 infection. T cell responses seem to be important in reducing disease severity and may mediate long-term protection against the virus [17–19]. Data coming from sever COVID-19 patients found an damaged function of CD4^+^T cells, associated with lower IFN-γ secretion [34]. In addition, vaccine-induced multi-protein specific T cell responses were largely preserved against the SARS-CoV-2 variant [35–37]. Recent studies proved inactivated SARS-CoV-2 vaccine-induced multi-protein specific T cell response against the Omicron variant and cross-recognition of the different variants by CD4^+^ and CD8^+^T-cells was maintained after booster vaccination [36, 37]. We observed activation of CD4^+^T and CD8^+^T cells at early state after prime and stonger after booster vaccination, indicating efficacy of priming T cells in eliciting cellular immunity against SARS-CoV-2. In addition, we found up to a half reductions of activated RBD-specific T cells at six months when compared to 40 days after the two dose of vaccine. This result was consistent with previously published article [38]. However, another study found the response to membrane and nucleoprotein remained largely unchanged after the third vaccination dose [35]. Importantly, vaccine-induced CD4^+^T cell responses to RBD protein were more prominent than CD8^+^T cell responses, in agreement with recent study [35]. Data showed substantial cross-reactive coronavirus T cells was observed in unexposed individuals [18, 39, 40]. Our result showed that RBD-specific CD4^+^T and CD8^+^T cells in 6% and 10% of unvaccinated individuals and a few cells expressed IL-2, TNF-α and IL-17A were detected, which may be indicate some degree of cross-reactivity and pre-existing immunity to SARS-CoV-2 RBD protein in some individuals.

In addition, we found CoronaVac vaccine induced a predominant Th1 response and a weak Th17 response on day 40 after prime immunization, and on day 60 after boost immunization, which was consistent with previously published reports [23, 38]. The ICS and ELISA results showed a high percentage of PBMC positive for Th1 cytokines IFN-γ, TNF-α, and IL-2 in vaccinated individuals, but a low percentage of expressed IL-4 associated with the Th2 response. However, these cytokines were not detected by 6 months after prime vaccination. This means that booster vaccination is important to prevent SARS-CoV-2 reinfection. Vaccine-induced polyfunctional T cells appear to have greater protective value. In our study, higher frequencies of multifunctional CD4 and CD8^+^T cells were observed on day 40 in prime vaccination and 60 in booster vaccination. Although the multifunctional RBD-specific T cells could wane over time, CD4^+^T cells co-expressing two cytokines were still detectable at six months following prime vaccination. These results demonstated that inactived vaccine induced a broad and robust CD4^+^T cell response to SARS-CoV-2 RBD protein, which may be contribut to long-term protective immunity.

Consistent with published study [38], we observed that booster vaccination effectively recalled specific antibodies responses to SARS-CoV-2 RBD protein, which had declined substantially 6 months after two doses of vaccination. In addition, the vaccine-induced IgG antibody was dominated by IgG1. Establishing immune memory is essential in the defense against SARS-CoV-2 infection [28]. The humoral immune response in our study confirmed that inactived vaccines induced a population of memory B cells that were durable for at least at six months after prime vaccination. Strikingly, the frequency of RBD-specific memory B cells that was focused on RBD significantly increased following booster vaccination, indicating that three dose of vaccines would be capable of rapidly producing functional antibodies against SARS-CoV-2 reinfection. Studies [9–12] have shown that a third dose of CoronaVac effectively recalled specific antibodies to SARS-CoV-2, which could be attributed to the durable memory B cell responses. In addition, we interrogated the correlation of CD4^+^T cell responses with CD8^+^T cell and humoral response. The notion that the functional role in protective immunity of CD4^+^T cell responses was proved by the correlation between CD4^+^T cells with CD8^+^T cell and humoral responses.

Cytokines coordinate the immune response was important to prevent systemic damage [41]. TNF-α and IL-17A were abundantly secreted cytokines in the 40d dose2 cohort and the 60d dose3 cohort. IL-12p70, a key cytokine that initiated the Th1 response, was remarkable increased following booster vaccination. These results were similar to ICS assay. The cytokine profile signatures of the vaccinated individuals in the 40d dose2 and 60d dose3 cohorts revealed several main clusters of correlations. Some of these cytokines play an auxiliary role in the proliferation of T and B cells. These results suggested prime and boost vaccination changed the cytokine signature of plasma. A broader and more complex cytokine pattern correlated with the dose of vaccination and the complexity of the cytokine correlation gradually weaken.

Our study has a few limitations. The sample size was small and the time points sampled in this study may not better detect the complete kinetics of the response of each immune component. Another limitation was that the study population generally tends to be young individuals. Therefore, the data may not complete represent the persistence of vaccine-induced immune response in elderly individuals. Finally, our study only measured S-RBD specific T and B cells responses. Additional studies will be required to detected other structural proteins including S, N and M due to non-S specific T and B cells have been shown to correlate with disease severity or protection.

## Conclusion

Collectively, these results demonstrated that CoronaVac vaccine induced protective immunities against SARS-CoV-2 virus, including durable memory B cells, strong and multifunctional CD4^+^T cell response with Th1 polarization. In addition, CoronaVac vaccine also induced a broader and complex cytokine pattern. The observation highlight the potential role of B cell and T cell responses in vaccine-induced long-term immunity, and will provide deeper insights into the potential immunobiological response of COVID-19 inactivated vaccines in humans and will be informative to the design of future vaccination strategies.

## Data Availability

This study-related data can be obtained from the corresponding author.
